# Global disparities in hepatocellular carcinoma care: insights from the ESGAR Global Abdominal Imaging Forum

**DOI:** 10.1007/s00330-026-12567-8

**Published:** 2026-06-09

**Authors:** Roberto Cannella, Federica Vernuccio, Cäcilia S. Reiner, Katja N. De Paepe, Giuseppe Brancatelli, Valérie Vilgrain, Marc Zins, Damian Tolan

**Affiliations:** 1 Department of Biomedicine, Neuroscience and Advanced Diagnostics (BiND), University of Palermo, Palermo, Italy; 2 Department of Radiology, Clinic Hirslanden Zurich, Zurich, Switzerland; 3 University of Zurich, Zurich, Switzerland; 4 Department of Radiology, Beth Israel Deaconess Medical Center, Harvard Medical School, Boston, USA; 5 Department of Radiology, Hôpital Beaujon, AP-HP. Nord, Clichy, France; 6 Centre de Recherche sur l’Inflammation, UMR1149, Université Paris Cité, Paris, France; 7 Department of Radiology, Saint Joseph and Marie Lannelongue Hospitals, Paris, France; 8 BioMedical Engineering and Imaging Institute, Icahn School of Medicine at MountSinai, 1470 Madison Ave, New York, NY 100292 USA; 9 Department of Radiology, Leeds Teaching Hospitals NHS Trust, Leeds, United Kingdom

**Keywords:** Carcinoma (hepatocellular), Developing countries, Health care infrastructure, Liver cirrhosis, Liver neoplasms

## Abstract

**Objectives:**

To analyze the intercontinental differences and inequities in the management of hepatocellular carcinoma (HCC), including surveillance, diagnosis, and treatment, through the survey responses from the participants of the Global Abdominal Imaging Forum on HCC, organized by the European Society of Gastrointestinal and Abdominal Radiology (ESGAR).

**Materials and methods:**

An online anonymous survey was distributed to the attendees of the Global Abdominal Imaging Forum on HCC. The survey consisted of 14 multiple-choice questions, covering demographic, epidemiological and occupational data, and information on the management of HCC. Global differences and differences between European and non-European countries were analyzed.

**Results:**

Of 1963 attendees from 110 countries, 408 (20.8%) from 67 countries responded to the survey. Multidisciplinary HCC meetings were reported with the highest frequency in North America (83.3%) and the lowest in Africa (41.2%). The most commonly reported tools for HCC surveillance were ultrasound (86.8%) and serum alpha-fetoprotein (79.4%). Global inequities were reported for contrast-enhanced ultrasound (*p* < 0.001, lowest access in South America, Africa, and Asia), MRI (*p* < 0.001, lowest access in Africa) and PET/CT (*p* < 0.001, lowest access in Africa). LI-RADS was the most common algorithm used for HCC diagnosis (83.6%), with the highest use reported in North America (94.4%). Heterogeneity in treatment availability was observed, with respondents from Africa reporting limited availability of surgery (41.2%), locoregional treatments (23.5%), liver transplantation (0%), and immunotherapy (17.6%).

**Conclusion:**

Although the surveillance strategy for HCC is similar at a global level, disparities exist in access to CEUS, MRI, PET/CT, and treatments for HCC.

**Key Points:**

***Question*** *What are the global disparities in hepatocellular carcinoma surveillance, diagnosis, and treatment reported by doctors working in different continents?*

***Findings*** *The survey from the Global Abdominal Imaging Forum on HCC revealed significant global variability with disparities regarding access to advanced imaging modalities and treatments, particularly affecting Africa.*

***Clinical relevance*** *Global efforts should focus on improving access to advanced imaging techniques (including MRI and PET/CT) and treatments (including transplantation and immunotherapy). Guidelines for the management of HCC should consider the existing regional disparities in healthcare infrastructure and resource availability to ensure more equitable HCC care worldwide.*

**Graphical Abstract:**

**Figure:**
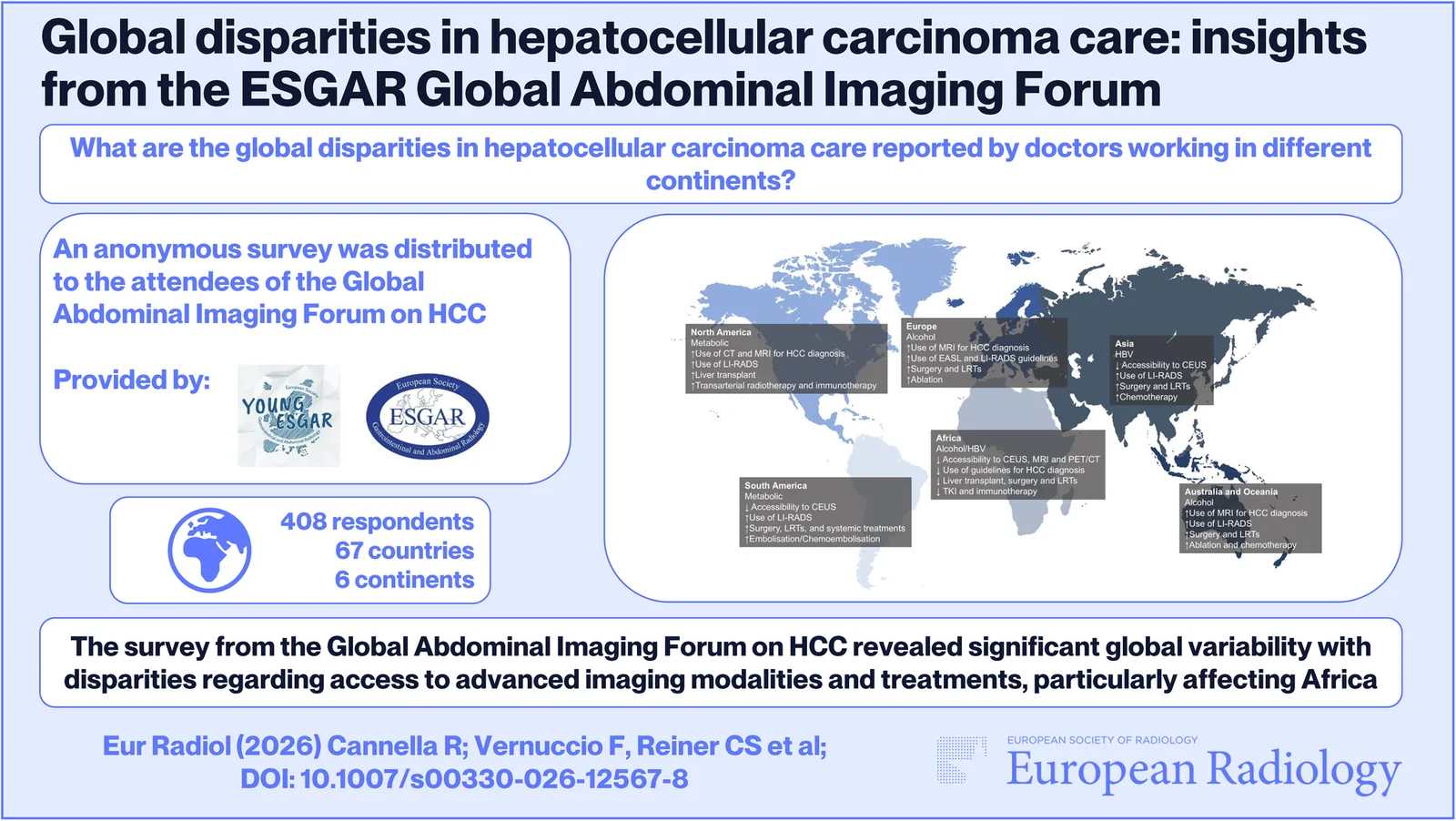

## Introduction

Hepatocellular carcinoma (HCC) is the most common primary malignancy of the liver, accounting for 85%–90% of all primary liver malignancies [1–3]. Cirrhosis and chronic hepatitis B are the predominant risk factors for hepatocellular carcinogenesis, although the steep rise of metabolic dysfunction-associated steatotic liver disease and the effective treatments for virus-related liver diseases are rapidly changing the epidemiology of chronic liver diseases and HCC [4]. The incidence of HCC cases is expected to increase worldwide by 55% in the next 20 years, due to the increasing age of the population and metabolic syndrome epidemics [5]. HCC remains a heterogeneous disease globally, owing to variations in epidemiological risk factors, screening and diagnostic approaches, and treatment strategies across different regions. This is reflected in the existence of multiple international and regional guidelines and clinical practice recommendations [6–10]. However, current differences in real-world management of HCC, including surveillance, diagnosis and treatment, have not been investigated globally, particularly in less developed countries.

The first Global Abdominal Imaging Forum on HCC was held on November 8, 2024. This event was organized by the European Society of Gastrointestinal and Abdominal Radiology (ESGAR), with the support of the following sister societies: Society of Abdominal Radiology (SAR), Abdominal Radiology Group of Australia and New Zealand (ARGANZ), Asian Society of Abdominal Radiology (ASAR), World Health Organization (WHO), the World Hepatitis Alliance, and Radiologists Without Borders. This was a free-of-charge 1-day global virtual event, live-streamed for 6 h, subtitled in five languages (English, Spanish, Portuguese, French, and Simplified Chinese), with speakers from six continents. All content was accessible on demand for the next 30 days and available in 32 languages. Following this event, participants were invited to contribute to a structured survey aimed at investigating global disparities in HCC management.

The aim of this study was to analyze the intercontinental differences and inequities in the management of HCC, including surveillance, diagnosis, and treatment, using the survey responses from the participants of the Global Abdominal Imaging Forum on HCC.

## Materials and methods

### Study design

Institutional Review Board approval was not required because the study did not collect any patient data or identifiable information. The study was conducted in accordance with the Strengthening the Reporting of Observational Studies in Epidemiology (STROBE) checklist [11].

An online survey was initially drafted by Young ESGAR members (Federica Vernuccio, Roberto Cannella, Katja De Paepe, and Cäcilia Reiner) and reviewed by the Global Abdominal Imaging Forum organizing committee (Damian Tolan, Giuseppe Brancatelli, Valérie Vilgrain, and Marc Zins). The survey was prepared in English, consisting of 14 multiple-choice questions (Supplementary material), including demographic, epidemiological, and occupational data (questions 1–5), as well as information on local management of HCC (questions 6–14). Multiple answers were allowed for: tests for HCC surveillance (question 7), guidelines used in clinical practice to report CT or MRI (question 10), available treatments for HCC (question 12), and available systemic therapies for HCC (question 14). Accessibility to imaging (question 8) was scored using a continuous 5-point scale, ranging from 1 (not accessible) to 5 (easily accessible).

### Survey administration

The survey was announced via email and was open to the attendees of the Global Abdominal Imaging Forum on HCC on November 7, 2024, and it was available until March 10, 2025. Regular reminders were sent via email by the ESGAR office to all attendees. The survey was distributed through the JotForm online platform (https://www.jotform.com/). Participation in the survey was voluntary. No personally identifiable information was recorded from any of the participants. To avoid missing data, all the questions were mandatory, with the exception of the country of origin, requiring the participants to provide at least one response to each question.

### Statistical analysis

Categorical variables are reported as numbers and percentages, while the continuous variable (accessibility of imaging) is provided as median and interquartile range. Responses were analyzed according to the self-declared continent of origin of the respondents (mandatory question) to compare global differences as well as differences between European and non-European countries. When the country of origin was available, responses were analyzed according to country income level, based on the World Bank Group country classification by income level [12]. Furthermore, responses were also analyzed according to the main practice location of the respondents. Categorical variables were compared with the Pearson χ^2^ test or Fisher’s exact test; the continuous variable (accessibility of imaging) was compared by using the Mann–Whitney U test or the Kruskal–Wallis test, as appropriate.

Statistical significance level was set at *p* < 0.05. Considering the exploratory nature of the analyses and the small number of survey questions, no adjustment for multiple comparisons was applied. Statistical analysis was performed with the IBM SPSS software version 26.0 (IBM Corp).

## Results

**Fig. 1:**
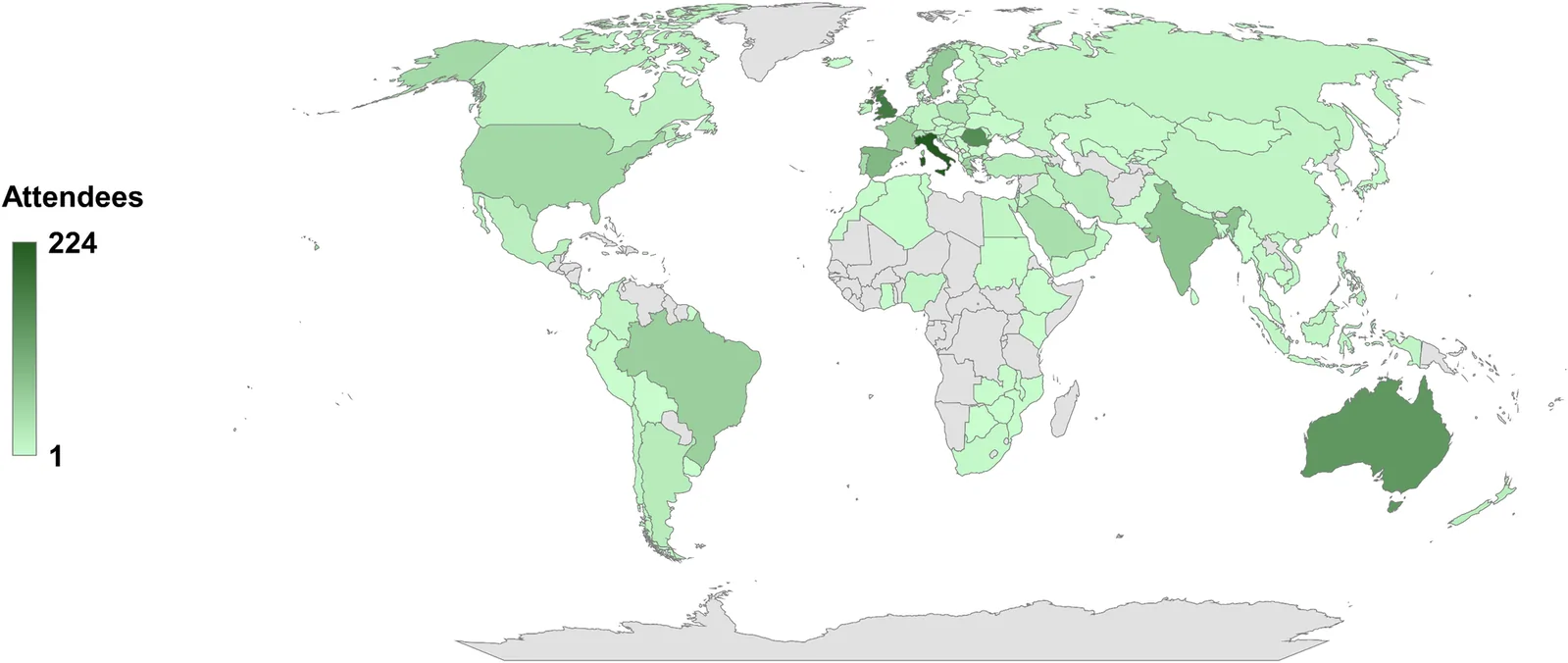
Graph with detailed distribution of attendees by countries

**Fig. 2:**
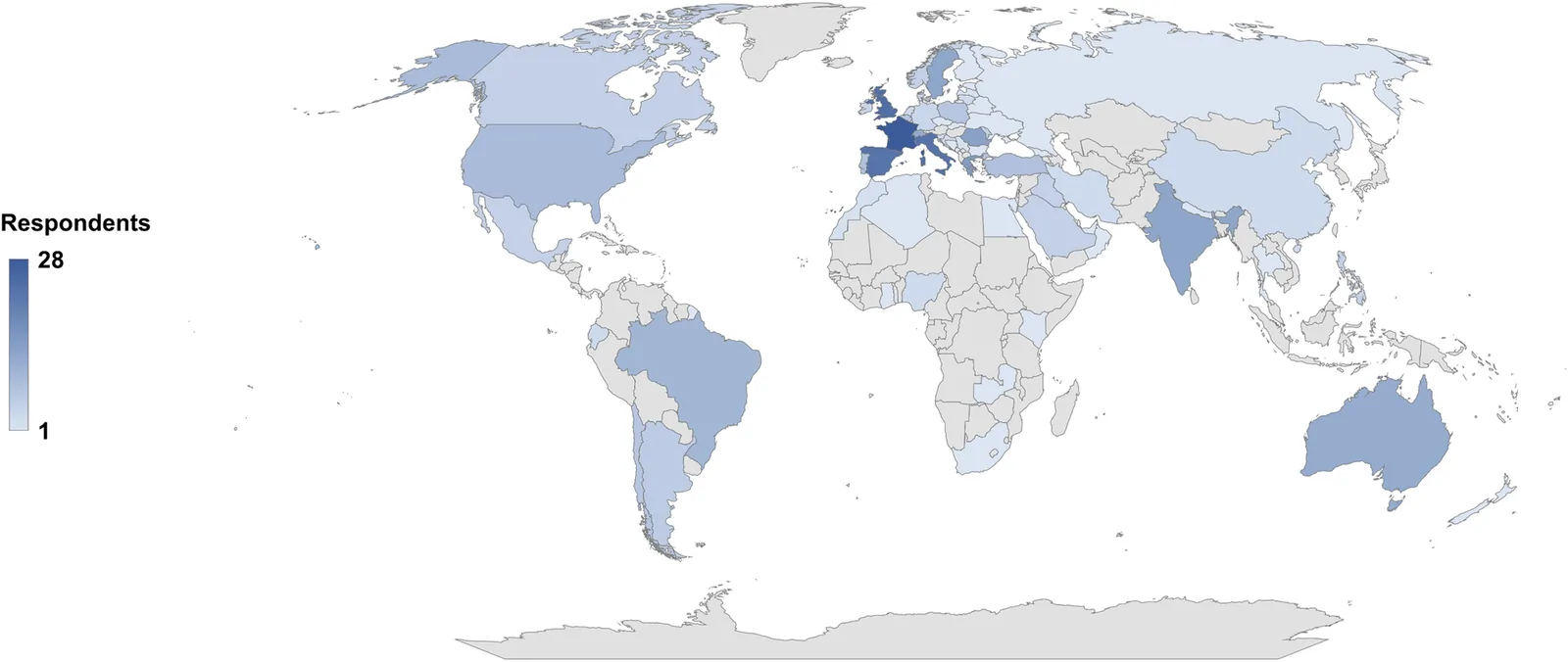
Distribution of respondents by countries

A total of 1963 people from 110 countries participated in the Global Abdominal Imaging Forum (Fig. 1). The top four participating countries in terms of number of attendees were Italy (*n* = 224, 11.4%), the United Kingdom (*n* = 176, 9.0%), Romania (*n* = 150, 7.6%), and Australia (*n* = 135, 6.9%). From the total number of attendees, 408 (20.8%) from 67 countries responded to the online survey (Fig. 2). General characteristics of the respondents are provided in Table 1. Most of the respondents were from Europe (*n* = 273, 66.9%). The top four participating countries in terms of number of respondents to the survey were France (*n* = 28, 6.9%), the United Kingdom (*n* = 24, 5.8%), Italy and Spain (*n* = 23, 5.6% each). Among the non-European countries, the highest number of responses was obtained from India (*n* = 13, 3.2%), Australia (*n* = 12, 2.9%), and Brazil (*n* = 10, 2.5%). Of note, 59 (14.5%) respondents did not report their country of origin. Of the 349 (85.5%) responses with a reported country of origin, 262 (75.1%) were from high-income countries, 56 (16.0%) from upper-middle-income countries, 29 (8.3%) from lower-middle-income countries, and 2 (0.6%) from low-income countries.

**Table 1:** General characteristics of the respondents

Characteristics	Number (%)
Continent	
Africa	17 (4.2)
Asia	53 (13.0)
Australia/Oceania	19 (4.7)
Europe	273 (66.9)
North America	18 (4.4)
South America	28 (6.9)
Profession	
Doctor/radiologists	307 (75.2)
Doctor—training	84 (20.6)
Radiographer/sonographer	16 (3.9)
Other	1 (0.2)
Medical specialty for doctors (*n* = 391)	
Radiology	376 (96.2)
Hepatology/gastroenterology	6 (1.5)
Oncology	5 (1.3)
General practice/family medicine	2 (0.5)
Surgery	2 (0.5)
Main practice location	
Community hospital—rural area	13 (3.2)
Community hospital—urban area	87 (21.3)
Government organization	29 (7.1)
Non-government organization	2 (0.5)
Private hospital	34 (8.3)
Private clinic	31 (7.6)
Retired/not employed	1 (0.2)
University/teaching hospital	208 (51.0)
Other	3 (0.7)

Categorical variables are expressed as numbers and percentages in parentheses

Overall, 307 (75.2%) of the respondents were specialized doctors and 84 (20.6%) were residents, with 376 (96.2%) practicing radiology, and 208 (51.0%) working in university/teaching hospitals. Particularly, respondents from Africa were most commonly residents (7/17, 41.2%) or doctors (6/17, 35.3%), working in university/teaching hospitals (9/17, 52.9%). There were no significant differences in the percentages of respondents working in university/teaching hospitals vs other practices across different continents (*p* = 0.675) and between European and non-European respondents (*p* = 0.310).

### Global differences in HCC epidemiology

**Fig. 3:**
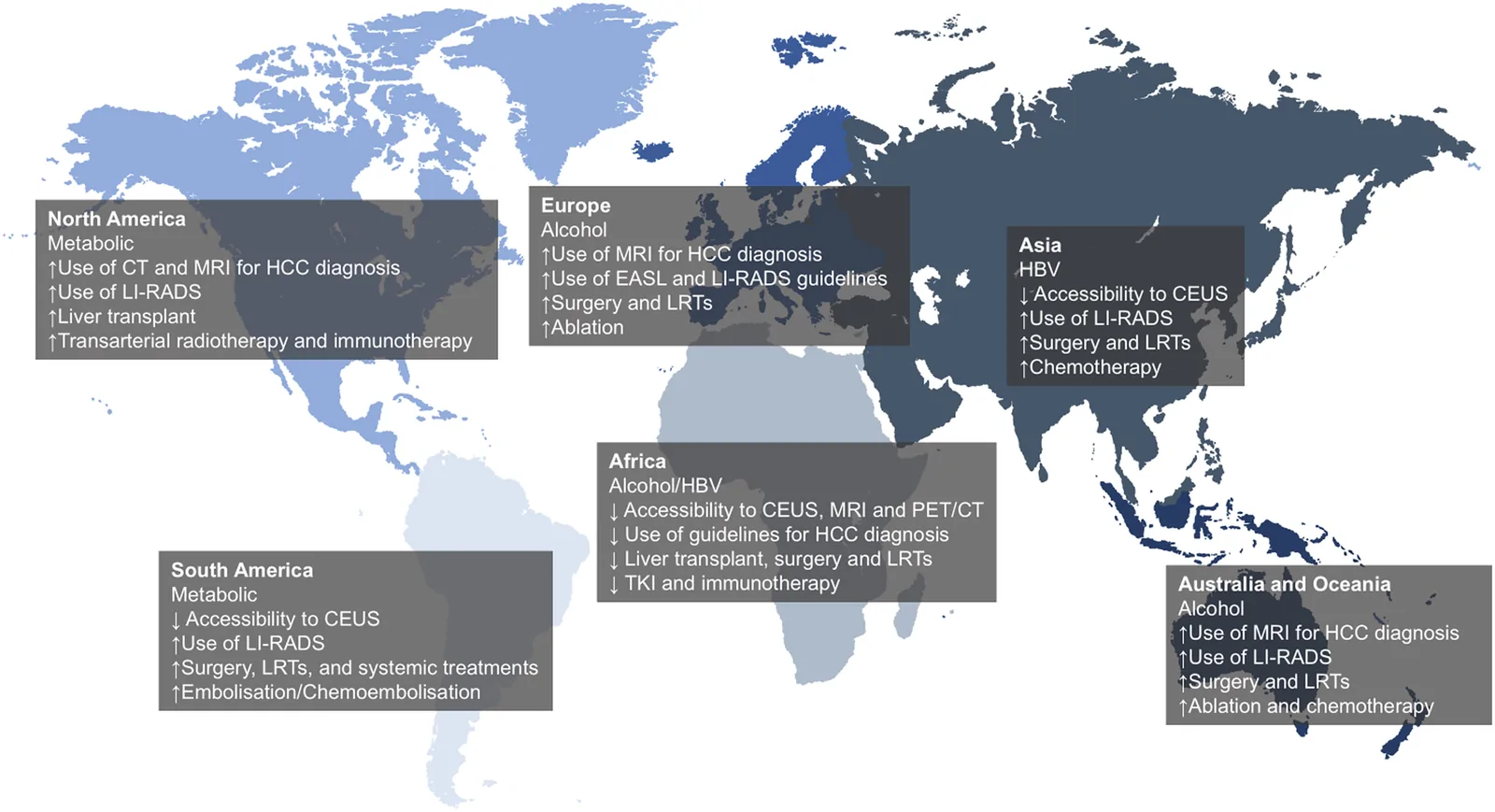
Main global differences according to the reported responses

Global differences in HCC care are analyzed in Table 2 and illustrated in Fig. 3. Differences in HCC according to the country income level and main practice location are provided in Supplementary Table 1 and Supplementary Table 2, respectively. The main causes of chronic liver disease varied significantly among continents (*p* < 0.001). Alcohol was reported as the leading cause in Europe (170/273, 62.3%) and in Australia and Oceania (10/19, 52.6%), while metabolic liver disease was predominant in North America (8/18, 44.4%) and South America (11/28, 39.3%). In Asia, hepatitis B was the most commonly reported cause (24/53, 45.3%).

**Table 2:** Global hepatocellular carcinoma practice and differences between European and non-European respondents

	All (*n* = 408)	Europe (*n* = 273)	North America (*n* = 18)	South America (*n* = 28)	Africa (*n* = 17)	Asia (*n* = 53)	Australia Oceania (*n* = 19)	*p*-value (all)	*p*-value (Europe vs non-Europe)
Commonest cause of chronic liver disease								**< 0.001**	**< 0.001**
Alcohol	216 (52.9)	170 (62.3)	6 (33.3)	10 (35.7)	7 (41.2)	13 (24.5)	10 (52.6)		
Hepatitis B	72 (17.6)	31 (11.4)	2 (11.1)	4 (14.3)	7 (41.2)	24 (45.3)	4 (21.1)		
Hepatitis C	52 (12.7)	38 (13.9)	1 (11.1)	3 (10.7)	3 (17.6)	4 (7.5)	2 (10.5)		
Metabolic	65 (15.9)	32 (11.7)	8 (44.4)	11 (39.3)	0 (0)	11 (20.8)	3 (15.8)		
Other	3 (0.7)	2 (0.7)	0 (0)	0 (0)	0 (0)	1 (1.9)	0 (0)		
Availability of multidisciplinary HCC meeting	305 (74.8)	208 (76.2)	15 (83.3)	20 (71.4)	7 (41.2)	40 (75.5)	15 (78.9)	**0.042**	0.343
Test for HCC surveillance*									
No surveillance	6 (1.5)	3 (1.1)	0 (0)	0 (0)	1 (5.9)	2 (3.8)	0 (0)	0.363	0.402
Alpha-fetoprotein	324 (79.4)	219 (80.2)	15 (83.3)	24 (85.7)	8 (47.1)	44 (83.0)	14 (73.7)	**0.027**	0.566
MRI	228 (55.9)	164 (60.1)	7 (38.9)	14 (50.0)	5 (29.4)	30 (56.6)	8 (42.1)	0.057	**0.015**
Ultrasound	354 (86.8)	229 (83.9)	18 (100)	25 (89.3)	15 (88.2)	49 (92.5)	18 (94.7)	0.189	**0.015**
Don’t know	6 (1.5)	5 (1.8)	0 (0)	0 (0)	1 (5.9)	0 (0)	0 (0)	0.508	0.668
Accessibility of imaging									
CEUS	2 (1, 4)	3 (1, 5)	2.5 (1, 5)	1 (1, 1)	1 (1, 1)	1 (1, 3)	2 (1, 3)	**< 0.001**	**< 0.001**
CT	5 (5, 5)	5 (5, 5)	5 (4.8, 5)	5 (4, 5)	5 (4, 5)	5 (5, 5)	5 (5, 5)	0.221	**0.016**
MRI	5 (4, 5)	5 (4, 5)	5 (4, 5)	4 (3, 5)	3 (2, 5)	5 (4, 5)	4 (3, 5)	**< 0.001**	**0.006**
PET/CT	3 (3, 5)	4 (3, 5)	4 (3, 4.3)	3 (1, 3)	1 (1, 2)	4 (3, 5)	3 (3, 5)	**< 0.001**	**0.026**
Ultrasound	5 (5, 5)	5 (5, 5)	5 (5, 5)	5 (5, 5)	5 (5, 5)	5 (5, 5)	5 (5, 5)	0.660	0.899
Commonest imaging to characterize a lesion suspicious for HCC								**< 0.001**	**0.019**
CEUS	8 (2.0)	4 (1.5)	2 (11.1)	3 (4.7)	3 (4.7)	3 (4.7)	3 (4.7)		
CT	162 (39.7)	98 (35.9)	8 (44.4)	15 (53.6)	12 (70.6)	24 (44.3)	5 (26.3)		
MRI	225 (55.1)	164 (60.1)	7 (38.9)	12 (42.9)	2 (11.8)	27 (50.9)	13 (68.4)		
PET/CT	2 (0.5)	0 (0)	0 (0)	0 (0)	1 (5.9)	0 (0)	1 (5.3)		
Ultrasound	11 (2.7)	7 (2.6)	1 (5.6)	0 (0)	1 (5.9)	2 (3.8)	0 (0)		
Guidelines used in clinical practice to report CT or MRI*									
APASL	3 (0.7)	1 (0.4)	0 (0)	0 (0)	0 (0)	2 (3.8)	0 (0)	0.166	0.255
EASL	68 (16.7)	65 (23.8)	1 (5.6)	0 (0)	0 (0)	1 (1.9)	1 (5.3)	**< 0.001**	**< 0.001**
KLCA-NCC	6 (1.5)	1 (0.4)	0 (0)	0 (0)	1 (5.9)	4 (7.5)	0 (0)	**0.002**	**0.017**
LI-RADS	341 (83.6)	226 (82.8)	17 (94.4)	27 (96.4)	6 (35.3)	47 (88.7)	18 (94.7)	**< 0.001**	0.538
Don’t know	17 (4.2)	9 (3.3)	0 (0)	2 (7.1)	3 (17.6)	3 (5.7)	0 (0)	0.056	0.211
No guidelines are used	48 (11.8)	32 (11.7)	1 (5.6)	0 (0)	9 (52.9)	6 (11.3)	0 (0)	**< 0.001**	0.969
Proportion of suspected HCC undergoing biopsy								**0.027**	0.085
No biopsies performed	34 (8.3)	21 (7.7)	2 (11.1)	5 (17.9)	1 (5.9)	2 (3.8)	3 (15.8)		
1–33%	230 (56.4)	144 (52.7)	15 (83.3)	13 (46.4)	12 (70.6)	31 (58.5)	15 (78.9)		
33–66%	90 (22.1)	67 (24.5)	0 (0)	7 (25.0)	1 (5.9)	15 (28.3)	0 (0)		
66–100%	54 (13.2)	41 (15.0)	1 (5.6)	3 (10.7)	3 (17.6)	5 (9.4)	1 (5.3)		
Available treatments for HCC*									
Liver transplant	272 (66.7)	189 (69.2)	15 (83.3)	22 (78.6)	0 (0)	31 (58.5)	15 (78.9)	**< 0.001**	
Locoregional treatments	355 (87.0)	241 (88.3)	18 (100)	25 (89.3)	4 (23.5)	50 (94.3)	17 (89.5)	**< 0.001**	0.118
Surgery/resection	364 (89.2)	247 (90.5)	18 (100)	25 (89.3)	7 (41.2)	50 (94.3)	17 (89.5)	**< 0.001**	0.278
Systemic therapy	350 (85.8)	237 (86.8)	18 (100)	28 (92.9)	9 (52.9)	45 (84.9)	15 (78.9)	**0.001**	0.243
Don’t know	26 (6.4)	16 (5.9)	0 (0)	2 (7.1)	7 (41.2)	1 (1.9)	0 (0)	**< 0.001**	0.397
Commonest locoregional treatment for HCC								**< 0.001**	**0.002**
Ablation	145 (35.5)	111 (40.7)	6 (33.3)	4 (14.3)	1 (5.9)	14 (26.4)	9 (47.4)		
Embolisation/chemoembolisation	171 (41.9)	107 (39.2)	8 (44.4)	19 (67.9)	2 (11.8)	30 (56.6)	5 (26.3)		
Radiotherapy—stereotactic	3 (1.7)	4 (1.5)	0 (0)	1 (3.6)	1 (5.9)	2 (3.8)	0 (0)		
Radiotherapy—transarterial/selective internal	8 (2.0)	5 (1.8)	3 (16.7)	1 (3.6)	0 (0)	4 (7.5)	3 (15.8)		
None	19 (4.7)	11 (4.0)	0 (0)	0 (0)	6 (35.3)	3 (3.8)	0 (0)		
Don’t know	49 (12.0)	35 (12.8)	1 (5.6)	3 (10.7)	8 (41.2)	1 (1.9)	2 (10.5)		
Available systemic therapies for HCC*									
Chemotherapy	279 (68.4)	175 (64.1)	15 (83.3)	16 (57.1)	9 (52.5)	49 (92.5)	15 (78.9)	**< 0.001**	**0.008**
Immunotherapy	243 (59.3)	163 (59.7)	15 (83.3)	14 (50.0)	3 (17.6)	35 (66.0)	13 (68.4)	**0.002**	0.931
Tyrosine kinase inhibitors	179 (43.9)	122 (44.7)	10 (55.6)	12 (42.9)	2 (11.8)	23 (43.4)	10 (52.6)	0.117	0.637
Anti-VEGF and immunotherapy	216 (52.9)	152 (55.7)	13 (72.2)	13 (46.4)	2 (11.8)	24 (45.3)	12 (63.2)	**0.003**	0.115
None	4 (1.0)	2 (0.7)	0 (0)	0 (0)	1 (5.9)	0 (0)	1 (5.3)	0.111	0.602
Don’t know	94 (23.0)	72 (26.4)	1 (5.6)	6 (21.4)	7 (41.2)	6 (11.3)	2 (10.5)	**0.017**	**0.025**

Continuous variables are expressed as medians and interquartile range (25th to 75th percentile) in parentheses, and it was compared using the Mann–Whitney U test or the Kruskal–Wallis test. Categorical variables are expressed as numbers and percentages in parentheses, and they are compared using the Pearson χ^2^ test or Fisher’s exact test

Statistically significant values (*p* < 0.05) are highlighted in bold

*APASL* Asia-Pacific Association for the Study of the Liver, *CEUS* contrast-enhanced ultrasound, *CT* computed tomography, *EASL* European Association for the Study of the Liver, *HCC* hepatocellular carcinoma, *KLCA-NCC* Korean Liver Cancer Association-National Cancer Center, *LI-RADS* Liver Imaging Reporting and Data System, *PET* positron emission tomography, *VEGF* Vascular Endothelial Growth Factor, *HBV* Hepatitis B Virus *LRTs* Locoregional Treatments

* Multiple answers were allowed

### Global differences in HCC surveillance and diagnosis

Globally, multidisciplinary HCC meetings were reported by 305 (74.8%) of respondents; the highest frequency was reported in North America (15/18, 83.3%) and the lowest in Africa (7/17, 41.2%). Multidisciplinary meetings were significantly more available in high-income countries (*p* < 0.001) and in university/teaching hospitals (*p* < 0.001). Ultrasound (US) and serum alpha-fetoprotein were indicated as the most common tools for HCC surveillance (86.8% and 79.4%, respectively). However, serum alpha-fetoprotein was reported by only 47.1% (8/17) of the respondents from Africa (*p* = 0.027). Regarding imaging access, global inequities were reported in the availability of contrast-enhanced ultrasound (CEUS) (*p* < 0.001, lower access in South America, Africa, and Asia), MRI (*p* < 0.001, lower access in Africa), and PET/CT (*p* < 0.001, lower access in Africa). However, CT and US were accessible on all continents. The accessibility of imaging was found to be significantly higher among European respondents compared to non-European respondents and among respondents from high-income countries for all the imaging modalities, with the exception of US. Accessibility of imaging was higher in university/teaching hospitals for CEUS (*p* < 0.001), MRI (*p* = 0.008), and PET/CT (*p* < 0.001). MRI was reported as the most commonly used imaging to characterize a suspicious lesion for HCC by 225 (55.1%) respondents, but with significant differences worldwide (*p* < 0.001), with 68.4% of respondents from Australia and Oceania, 60.1% from Europe, but only 11.8% (2/17) from Africa using MRI as the main imaging modality. LI-RADS was reported as the most common algorithm used for HCC diagnosis (83.6% of the global responses), with the highest usage reported in North America (17/18, 94.4%), South America (27/28, 96.4%), Australia and Oceania (18/19, 94.7%), and the lowest in Africa (6/17, 35.5%). EASL guidelines were mostly used in Europe (65/273, 23.8%, *p* < 0.001), while the KLCA-NCC had the highest reported frequency in Asia (7/53, 7.5%, *p* = 0.002). Globally, 56.4% of the respondents stated that biopsy is performed in 1–33% of suspected HCC lesions. However, significant differences were observed in the reported proportion of biopsies for suspected HCC (*p* = 0.027), ranging from 83.3% of respondents in North America to 52.7% in Europe who reported performing biopsies in 1–33% of suspected HCC.

### Global differences in HCC treatment

Globally, surgery was the most available treatment option (89.2%), followed by locoregional treatments (87.0%) and systemic therapy (85.8%). However, inequities in treatment availability were observed. Liver transplantation was more available for respondents from North America (15/18, 83.3%), while no respondents from Africa reported availability of liver transplantation (*p* < 0.001). Respondents from Africa also reported lower availability of surgery (7/17, 41.2%) and locoregional treatments (4/17, 23.5%). High-income countries had significantly higher availability of liver transplantation (*p* < 0.001), surgery (*p* = 0.030), and locoregional treatments (*p* = 0.013). All treatments for HCC were more commonly available in university/teaching hospitals (*p* < 0.001).

Transarterial (chemo)embolisation was the most commonly available locoregional treatment globally (41.9%), followed by ablation (35.5%), with significant differences worldwide. Ablation had the highest reported frequency in Australia and Oceania (9/19, 47.7%), transarterial (chemo)embolisation in Asia (30/53, 56.6%), and transarterial radioembolisation in North America (3/18, 16.7%). Regarding available systemic treatments, chemotherapy was reported as the most frequently available systemic treatment worldwide (68.4%), with the highest reported frequency in Asia (49/53, 92.5%, *p* < 0.001). Respondents from Africa reported the lowest frequencies of immunotherapy (3/17, 17.6%), tyrosine kinase inhibitors (2/17, 11.8%), and anti-VEGF and immunotherapy (2/17, 11.8%). All the systemic treatments were more frequently available in university/teaching hospitals.

## Discussion

The Global Abdominal Imaging Forum on HCC, organized by ESGAR and its sister societies, aimed at discussing current global practices and identifying gaps and management challenges for HCC. The analysis of responses from its participants has demonstrated significant health inequalities, including accessibility to important diagnostic imaging modalities and treatments. With the exception of US, European respondents reported higher accessibility to all imaging modalities, with MRI being the most commonly used modality for characterizing suspected HCC lesions. In North America, respondents reported the highest frequency of multidisciplinary meetings and the greatest availability of liver transplantation. In contrast, despite the small sample size, respondents from Africa highlighted limited use of multidisciplinary meetings, low accessibility to CEUS, MRI, and PET/CT, and limited availability of liver transplantation, locoregional treatments, surgery, and immunotherapy. Similar differences were observed according to the country’s income level. These results reflect wider inequities in global healthcare infrastructure, access to advanced imaging modalities, and cost-related issues for the treatments for HCC.

In agreement with current guidelines, US was reported as the most common test for HCC surveillance worldwide, followed by serum alpha-fetoprotein [13]. Although both the American Association for the Study of Liver Diseases (AASLD) and the European Association for the Study of the Liver (EASL) recommend US as the primary modality for HCC surveillance, the addition of alpha-fetoprotein is recommended by the AASLD, whereas the EASL considers its use optional [6, 8]. In the current survey, 60.1% of respondents from Europe reported using MRI for HCC surveillance. Possible explanations for this trend include growing interest in abbreviated MRI protocols for HCC surveillance and the increased incidence of metabolic dysfunction-associated steatotic liver disease in Western countries, where US may be non-diagnostic [14]. Moreover, the sensitivity of US has been reported to be low for detecting early-stage HCC, and it can be influenced by the patient’s body habitus as well as the underlying etiology of chronic liver disease [15]. While studies on abbreviated MRI have shown improved sensitivity for the early HCC detection in selected patients with poor visualization of the liver on US, the exam lengths, costs, contraindications, and availability of MRI scanners suggest that MRI may be unfeasible for HCC surveillance globally [16–18]. Therefore, its use is not yet supported by current guidelines [6, 8].

The current survey also highlights inequities in the accessibility of different imaging modalities worldwide. While US and CT were reported to be easily accessible in all continents, CEUS was reported to be less accessible in South America, Africa, and Asia, with less access to MRI and PET/CT in Africa. These results echo global data, with Africa having the lowest number of MRI units per million inhabitants worldwide, which is fewer than 0.7 scanners per million inhabitants in most African countries [19]. A survey in West Africa reported 84 MRI units serving more than 372 million inhabitants, with some countries having no MRI scanners [20]. By contrast, a recent study from the European Union Radiation, Education, Staffing & Training (EU-REST) project reported 7736 MRI scanners in Europe, with an average of 16 MRIs per million inhabitants, reaching up to 30 MRIs per million inhabitants in Greece [21]. Similarly, the limited availability of US contrast agents represents the main challenge for the use of CEUS in low-income countries. Notably, the majority of Asian respondents were from India, whereas no responses were obtained from the Republic of Korea or Japan, in which CEUS is widely available. This could explain the low accessibility of CEUS reported in the current survey, which could have been affected by the intra-continental heterogeneity. Global efforts should focus on equitable access to CEUS and MRI in those areas with limited availability to improve earlier diagnosis, treatment, and outcomes in patients with HCC and reduce global disparities.

LI-RADS was the most common classification system for reporting CT and MRI globally, with the highest usage reported in North America (96.4%). A recent survey on non-academic radiologists, mostly from the United States and Canada, reported that LI-RADS was adopted by 73.6% of the respondents [22]. That study identified lack of familiarity (25.1%), lack of use by referring clinicians (21.6%), and perceived complexity (14.5%) as the main barriers for LI-RADS adoption [22]. Conversely, a German survey showed that 26% of respondents used LI-RADS, with the main barriers for its use being the lack of experience (30.6%) and lack of practicability (6.5%) [23]. Our survey also showed that the EASL guidelines were most commonly adopted in Europe. However, the 2025 update of the EASL Clinical Practice Guidelines on the management of HCC endorsed the use of the LI-RADS algorithm for imaging, reporting, and noninvasive diagnosis of HCC [6]. This major update represents a significant advancement toward a global standardization and unification of the HCC diagnosis under the LI-RADS diagnostic criteria [24], but clearly, further effort is needed to implement standardization and LI-RADS adoption in non-Western countries.

The treatment options for HCC offered in respondents centers showed significant inequities worldwide. Liver transplantation was reported more frequently by respondents from North America, while those from Africa reported no availability of liver transplantation, and limited availability of surgery and locoregional treatments. While general recommendations for HCC management are similar across current guidelines, limited accessibility, costs, and local expertise likely account for the observed global disparities. There is a wide global variability in access to liver transplantation, with the United States performing the highest volume of liver transplantations worldwide, reaching more than ten thousand liver transplantations in 2023 [25]. However, HCC accounted for only 10.4% of liver transplantation indications in the United States according to the OPTN/SRTR 2023 Annual Data Report [25]. Limited data are available from Africa and Middle-Eastern countries regarding the number of liver transplantations [26]. The current survey also underlines the heterogeneity of locoregional and systemic treatments for HCC. Ablation and transarterial (chemo)embolization were reported as the most commonly performed locoregional treatments, but their availability varied significantly, being the lowest in Africa. The most commonly reported systemic treatments were chemotherapy and immunotherapy. According to the latest Barcelona Clinic Liver Cancer (BCLC) treatment recommendation, immunotherapy (particularly the combination of Atezolizumab-Bevacizumab or Durvalumab-Tremelimumab) should be considered as the first-line treatment in patients with advanced HCC [27], but access to immunotherapy in our survey was highest in North America and lowest in Africa. Despite several combination treatments under investigation for the treatment of advanced HCC, the costs of immunotherapy remain much higher compared to the tyrosine kinase inhibitors [28, 29]. These variations in treatment accessibility may contribute to global differences in HCC outcomes, with the highest mortality observed in South-Eastern Asia and sub-Saharan Africa [5].

Limitations are recognized in this study. First, the number of respondents from some countries was low (overall response rate of 20.8%), especially from Africa, nonetheless reflecting the distribution of the attendees of the Global Abdominal Imaging Forum. Furthermore, the 51.0% of the respondents reported working in university/teaching hospitals, while only 3.2% practiced in rural community hospitals. This may represent a source of bias for the survey, likely related to the composition of the live Global Imaging Forum audience and to the technological barriers associated with participation in the event and the survey. Language barriers might also have contributed because the survey was produced in English only, while the event itself was subtitled in five languages. Country of origin was not specified by 14.5% of the respondents, limiting the analysis by country income level to the subset of responses with reported country of origin. The comparison of different continents could also have been affected by the incomplete representation of all countries and the unequal responses among them. Finally, respondents were limited to the people attending the 2024 Global Abdominal Imaging Forum on HCC, which may introduce a selection bias. This could also explain the high proportion of participants working in universities or teaching hospitals. While a broader audience would be able to capture more precise data on the HCC current practice, these results nonetheless reflect the perspectives of radiologists and clinicians specifically interested in HCC from different regions of the world.

In conclusion, this survey of international radiologists from the Global Imaging Forum on HCC is the largest of its kind and highlights continuing global disparities in the diagnosis and treatment of HCC, despite the small sample of responses obtained from Africa. International healthcare policy must focus on improving access to advanced imaging modalities (such as MRI and PET/CT) and treatments (including transplantation and immunotherapy) to address inequity in resource provision, particularly in Africa. Future collaboration among scientific societies from different regions will be crucial to overcome barriers related to global inequities. Without a deliberate focus, the outcomes for patients with HCC are likely to remain poor in low-income countries.

## Supplementary information

Supplementary information

## Abbreviations

CEUS: Contrast-enhanced ultrasound
ESGAR: European Society of Gastrointestinal and Abdominal Radiology
HCC: Hepatocellular carcinoma
LI-RADS: Liver Imaging Reporting and Data System
MRI: Magnetic resonance imaging
PET/CT: Positron emission tomography–computed tomography
