# Supplementary material for: Global disparities in hepatocellular carcinoma care: insights from the ESGAR Global Abdominal Imaging Forum
Source: Eur Radiol. 2026 Jun 9;36(10):7988–97. doi: 10.1007/s00330-026-12567-8 (PMC13574859; doi:10.1007/s00330-026-12567-8)
Supplement: Supplementary file 1 [file 330_2026_12567_MOESM1_ESM.pdf]

Global Disparities in Hepatocellular Carcinoma Care: Insights from the ESGAR Global Abdominal Imaging Forum
Electronic Supplementary Materials

HCC survey questions:

1. In which continent do you practice?
- Africa
- Asia
- Australasia/Oceana
- Europe
- North America
- South America

Supplementary – which country? (free text)

2. What is your Profession?
- Doctor - Independent clinical practice
- Doctor - Training
- Medical Student
- Healthcare administration
- Nursing
- Pharma/Industry
- Radiographer
- Other: (free text)____________

3. If you are a doctor - what is your specialty?
- General Medicine
- General Practice/Family Medicine
- Hepatology/Gastroenterology
- Public health
- Oncology
- Radiology
- Surgery
- Other:(free text)____________

4. Where is your main practice location?
- Community hospital –rural area
- Community hospital - urban area
- Government organization
- Non-Government organization
- Private Hospital
- Private Clinic
- Retired/Not employed
- University/Teaching Hospital
- Other - (free text)

5. What is the commonest cause of chronic liver disease in your patient population?
- Alcohol

- Hepatitis B
- Hepatitis C
- Metabolic
- Other: (free text)

6. Does your department have a multidisciplinary team meeting to discuss HCC patient management?
- No
- Yes

7. Which tests are performed for HCC surveillance in your practice? (Tick all that apply)
- No surveillance performed
- Alpha Fetoprotein
- Magnetic resonance imaging
- Ultrasound
- Don't know

8. How accessible are these imaging modalities for HCC for your patients (1=not accessible, 3 = quite accessible, 5 = easily accessible)?
- Contrast Enhanced Ultrasound (1-5)
- CT (1-5)
- MRI (1-5)
- PET/CT (1-5)
- Ultrasound (1-5)
- Don't know

9. What technique is the commonest to characterize lesions suspicious for HCC in your practice?
- Contrast Enhanced Ultrasound
- CT
- MRI
- PET/CT
- Ultrasound
- Don't know

10. Which guidelines are used in your practice to report CT or MRI in patients at risk of HCC? (Tick all that apply)
- APASL
- EASL
- KLCA-NCC
- LI-RADS
- Don't know
- No guidelines are used

11. Estimate the proportion of liver lesions suspicious of HCC in your practice which undergo biopsy?
- No biopsies are performed
- 1-33%
- 33-66%
- 66-100%

12. What treatments are available for your patients with HCC? (Tick all that apply)
- Liver Transplant

- Locoregional treatment
- Surgery/Resection
- Systemic therapy
- Don't know

13. Which is the commonest locoregional treatment for HCC in your hospital/practice?

- Ablation
- Embolisation/Chemoembolisation
- Radiotherapy – stereotactic
- Radiotherapy – transarterial/selective internal
- None
- Don't know

14. Which systemic therapies are available for your patients with HCC patients (include all the options that apply)?

- Chemotherapy
- Immunotherapy
- Tyrosine kinase inhibitors
- Anti-VEGF and immunotherapy (atezolizumab + bevacizumab)
- None
- Don't know

Supplementary Table 1: Differences in hepatocellular carcinoma care according to the country income level.

	High-income counties (*n* = 262)	Others (*n* = 87)	p-value
**Commonest cause of chronic liver disease**			**< 0.001**
Alcohol	158 (60.3)	25 (28.7)	
Hepatitis B	28 (10.7)	33 (37.9)	
Hepatitis C	36 (13.7)	11 (12.6)	
Metabolic	38 (14.5)	17 (19.5)	
Other	2 (0.8)	1 (1.1)	
**Availability of multidisciplinary HCC meeting**	209 (79.8)	55 (63.2)	**0.002**
**Test for HCC surveillance***			
No surveillance	3 (1.1)	3 (3.4)	0.167
Alpha Fetoprotein	211 (80.5)	66 (75.9)	0.351
MRI	139 (53.1)	50 (57.5)	0.474
Ultrasound	230 (87.8)	76 (87.4)	0.916
Don't know	3 (1.1)	0 (0)	0.577
**Accessibility of imaging**			
CEUS	3 (1, 5)	1 (1, 2)	**< 0.001**
CT	5 (5, 5)	5 (5, 5)	**0.028**
MRI	5 (4, 5)	4 (3, 5)	**0.008**
PET/CT	4 (3, 5)	3 (2, 4)	**0.007**
Ultrasound	5 (5, 5)	5 (5, 5)	0.419
**Commonest imaging to characterize a lesion suspicious for HCC**			**0.032**
CEUS	4 (1.5)	3 (3.4)	
CT	96 (36.6)	44 (50.6)	
MRI	155 (59.2)	35 (40.2)	
PET/CT	1 (0.4)	1 (1.1)	

Ultrasound	6 (2.3)	4 (4.6)	
**Guidelines used in clinical practice to report CT or MRI***			
APASL	2 (0.8)	2 (2.3)	1.000
EASL	58 (22.1)	4 (4.6)	**< 0.001**
KLCA-NCC	2 (0.8)	3 (1.5)	0.260
LI-RADS	223 (85.1)	74 (85.1)	0.990
Don't know	7 (2.7)	4 (4.6)	0.477
No guidelines are used	26 (9.9)	12 (13.8)	0.315
**Proportion of suspected HCC undergoing biopsy**			0.816
No biopsies performed	25 (9.5)	7 (8.0)	
1-33%	152 (58.0)	47 (54.0)	
33-66%	50 (19.1)	19 (21.8)	
66-100%	35 (13.4)	14 (16.1)	
**Available treatments for HCC***			
Liver transplant	190 (72.5)	45 (51.7)	**< 0.001**
Locoregional treatments	237 (90.5)	70 (80.5)	**0.013**
Surgery/Resection	241 (92.0)	73 (83.9)	**0.030**
Systemic therapy	234 (89.3)	71 (81.6)	0.061
Don't know	12 (4.6)	9 (10.3)	0.050
**Commonest locoregional treatment for HCC**			**0.011**
Ablation	102 (38.9)	17 (19.5)	
Embolisation/Chemoembolisation	106 (40.5)	47 (54.0)	
Radiotherapy – stereotactic	3 (1.1)	4 (4.6)	
Radiotherapy – transarterial/selective internal	10 (3.8)	2 (2.3)	
None	12 (4.6)	5 (5.7)	
Don't know	29 (11.1)	12 (13.8)	
**Available systemic therapies for HCC***			
Chemotherapy	174 (66.4)	67 (77.0)	0.064
Immunotherapy	168 (64.1)	47 (54.0)	0.093
Tyrosine kinase inhibitors	136 (51.9)	28 (32.2)	**0.001**
Anti-VEGF and immunotherapy	160 (61.1)	33 (37.9)	**< 0.001**
None	3 (1.1)	1 (1.1)	1.000
Don't know	58 (22.1)	16 (18.4)	0.459

Note.—Continuous variables are expressed as medians and interquartile range (25th to 75th percentile) in parenthesis, and they were compared with the Pearson χ2 test or Fisher's exact test. Categorical variable (accessibility of imaging) was expressed as numbers and percentages in parenthesis, and it was compared with the Mann-Whitney U test. *Multiple answers were allowed. Statistically significant values (p <0.05) are highlighted in bold.

Abbreviations: APASL: Asia-Pacific Association for the Study of the Liver; CEUS: contrast-enhanced ultrasound; CT: computed tomography; EASL: European Association for the Study of the Liver; HCC: hepatocellular carcinoma; KLCA-NCC: Korean Liver Cancer Association-National Cancer Center LI-RADS: Liver Imaging Reporting and Data System; PET: positron emission tomography.

Supplementary Table 2: Differences in hepatocellular carcinoma care between university/teaching hospitals and other practice locations.

	University/Teaching Hospital (*n* = 208)	Others (*n* = 200)	p-value
**Commonest cause of chronic liver disease**			0.225
Alcohol	110 (52.9)	106 (53.0)	

Hepatitis B	31 (14.9)	41 (20.5)	
Hepatitis C	30 (14.4)	22 (11.0)	
Metabolic	34 (16.3)	31 (15.5)	
Other	3 (1.4)	0 (0)	
**Availability of multidisciplinary HCC meeting**	187 (89.9)	118 (59.0)	**< 0.001**
**Test for HCC surveillance***			
No surveillance	1 (0.5)	6 (2.5)	0.116
Alpha Fetoprotein	172 (82.7)	152 (76.0)	0.095
MRI	120 (57.7)	108 (54.0)	0.453
Ultrasound	183 (88.0)	171 (85.5)	0.460
Don’t know	2 (1.0)	4 (2.0)	0.441
**Accessibility of imaging**			
CEUS	3 (1, 5)	2 (1, 3)	**< 0.001**
CT	5 (5, 5)	5 (5, 5)	0.148
MRI	5 (4, 5)	4 (3.5, 5)	**0.008**
PET/CT	4 (3, 5)	3 (2, 4)	**< 0.001**
Ultrasound	5 (5, 5)	5 (5, 5)	0.890
**Commonest imaging to characterize a lesion suspicious for HCC**			0.076
CEUS	3 (1.4)	5 (2.5)	
CT	71 (34.1)	91 (45.5)	
MRI	129 (62.0)	96 (62.0)	
PET/CT	1 (0.5)	1 (1.5)	
Ultrasound	4 (1.9)	7 (3.5)	
**Guidelines used in clinical practice to report CT or MRI***			
APASL	2 (1.0)	1 (0.5)	1.000
EASL	44 (21.2)	24 (12.0)	**0.013**
KLCA-NCC	3 (1.4)	3 (1.5)	1.000
LI-RADS	180 (86.5)	161 (80.5)	0.100
Don’t know	5 (2.4)	12 (6.0)	0.069
No guidelines are used	19 (9.1)	29 (14.5)	0.093
**Proportion of suspected HCC undergoing biopsy**			**0.001**
No biopsies performed	8 (3.8)	26 (13.0)	
1-33%	132 (63.5)	98 (49.0)	
33-66%	40 (19.2)	50 (25.0)	
66-100%	28 (13.5)	26 (13.0)	
**Available treatments for HCC***			
Liver transplant	159 (76.4)	113 (56.5)	**< 0.001**
Locoregional treatments	194 (93.3)	161 (80.5)	**< 0.001**
Surgery/Resection	197 (94.7)	167 (83.5)	**< 0.001**
Systemic therapy	197 (94.7)	153 (76.6)	**< 0.001**
Don’t know	3 (1.4)	23 (11.5)	**< 0.001**
**Commonest locoregional treatment for HCC**			**<0.001**
Ablation	80 (38.5)	65 (32.5)	
Embolisation/Chemoembolisation	89 (42.8)	82 (41.0)	
Radiotherapy – stereotactic	5 (2.4)	3 (1.5)	
Radiotherapy – transarterial/selective internal	15 (7.2)	1 (0.5)	
None	6 (2.9)	3 (1.5)	
Don’t know	13 (6.3)	36 (18.0)	
**Available systemic therapies for HCC***			
Chemotherapy	157 (75.5)	122 (61.0)	**0.002**
Immunotherapy	142 (68.3)	101 (50.5)	**< 0.001**
Tyrosine kinase inhibitors	122 (58.7)	57 (28.5)	**< 0.001**
Anti-VEGF and immunotherapy	141 (67.8)	75 (37.5)	**< 0.001**
None	0 (0)	4 (2.0)	0.057

Don’t know	30 (14.4)	64 (32.0)	**< 0.001**

Note.—Continuous variables are expressed as medians and interquartile range (25th to 75th percentile) in parenthesis and they were compared with the Pearson χ2 test or Fisher’s exact test. Categorical variable (accessibility of imaging) was expressed as numbers and percentages in parenthesis and it was compared with the Mann-Whitney U test. *Multiple answers were allowed. Statistically significant values (*p* <0.05) are highlighted in bold.

Abbreviations: APASL: Asia-Pacific Association for the Study of the Liver; CEUS: contrast-enhanced ultrasound; CT: computed tomography; EASL: European Association for the Study of the Liver; HCC: hepatocellular carcinoma; KLCA-NCC: Korean Liver Cancer Association-National Cancer Center LI-RADS: Liver Imaging Reporting and Data System; PET: positron emission tomography.
